# Do Low Molecular Weight Agents Cause More Severe Asthma than High Molecular Weight Agents?

**DOI:** 10.1371/journal.pone.0156141

**Published:** 2016-06-09

**Authors:** Olga Meca, María-Jesús Cruz, Mónica Sánchez-Ortiz, Francisco-Javier González-Barcala, Iñigo Ojanguren, Xavier Munoz

**Affiliations:** 1 Servicio de Neumología, Hospital General Universitario Morales Messeguer, Murcia, Spain; 2 Servicio de Neumología, Hospital Universitario Vall d’Hebron, Barcelona, Catalonia, Spain; 3 Centro de Investigación en Red de Enfermedades Respiratorias (CIBERES), Instituto de Salud Carlos III (ISCIII), Barcelona, Spain; 4 Respiratory Department, Clinic University Hospital, Santiago de Compostela, Spain; 5 Department of Cell Biology, Physiology and Immunology, Universitat Autònoma de Barcelona, Catalonia, Spain; North Carolina State University, UNITED STATES

## Abstract

**Introduction:**

The aim of this study was to analyse whether patients with occupational asthma (OA) caused by low molecular weight (LMW) agents differed from patients with OA caused by high molecular weight (HMW) with regard to risk factors, asthma presentation and severity, and response to various diagnostic tests.

**Methods:**

Seventy-eight patients with OA diagnosed by positive specific inhalation challenge (SIC) were included. Anthropometric characteristics, atopic status, occupation, latency periods, asthma severity according to the Global Initiative for Asthma (GINA) control classification, lung function tests and SIC results were analysed.

**Results:**

OA was induced by an HMW agent in 23 patients (29%) and by an LMW agent in 55 (71%). A logistic regression analysis confirmed that patients with OA caused by LMW agents had a significantly higher risk of severity according to the GINA classification after adjusting for potential confounders (OR = 3.579, 95% CI 1.136–11.280; p = 0.029). During the SIC, most patients with OA caused by HMW agents presented an early reaction (82%), while in patients with OA caused by LMW agents the response was mainly late (73%) (p = 0.0001). Similarly, patients with OA caused by LMW agents experienced a greater degree of bronchial hyperresponsiveness, measured as the difference in the methacholine dose-response ratio (DRR) before and after SIC (1.77, range 0–16), compared with patients with OA caused by HMW agents (0.87, range 0–72), (p = 0.024).

**Conclusions:**

OA caused by LMW agents may be more severe than that caused by HMW agents. The severity of the condition may be determined by the different mechanisms of action of these agents.

## Introduction

The term”work-related asthma” encompasses both occupational asthma (OA) and work-exacerbated asthma (WEA) [1]. OA is characterized by variable airflow limitation and/or hyperresponsiveness and/or inflammation due to causes and conditions attributable to a particular occupational environment and not to stimuli encountered outside the workplace [2], while WEA is defined as the aggravation of pre-existing or coincident (adult new-onset) asthma due to workplace environmental exposure [3]. OA is subdivided into immunological or non-immunological forms, with the reactive airway dysfunction syndrome (RADS) being the most characteristic example of the latter presentation [4]. A recent evidence-based review of the literature identified 372 causative agents of immunological asthma and 184 different causes of irritant or non-immunological OA [5].

Immunological OA, caused by workplace sensitizers, is characterized by the appearance of work-related asthma symptoms after a latency period. The causative agent may be either a high or a low molecular weight agent (HMW and LMW, respectively). HMW agents are protein-derived antigens and are generally considered to cause sensitization through an IgE-mediated mechanism and so allergy skin-prick test and measurements of allergen-specific antibodies can aid diagnosis [6]. Although specific IgE antibodies have also been detected in OA induced by some LMW agents [7], and several studies have suggested that immunologic mechanisms are involved in LMW-related OA [8–9], the exact mechanisms have not yet been fully characterized. In fact, the few studies carried out to date have demonstrated variable patterns of response to HMW and LMW agents; in the main, HMW agents seem to induce early or dual asthmatic reactions, while LMW agents produce delayed reactions [10–11].

It is not known whether differences in the pathogenesis of immunological OA also lead to differences in clinical presentation. Early studies in the 1990s suggested that the natural history of the onset of OA varies according to the sensitizing agent, and that factors such as age, gender, atopy, rhinitis, and smoking habit might influence the development of the condition [10]. However, recent studies suggest that the intensity of exposure may affect the risk of development of symptoms and sensitization more than host markers or the type of agent [12]. Whether or not the severity of asthma is related to the type of causal agent is also unclear.

The objective of this study was to analyse the differences in relation to possible risk factors, asthma presentation and severity, and response to various diagnostic tests in patients with OA caused by either HMW or LMW agents.

## Patients and Methods

### Type of study

Retrospective study using data from medical charts, conducted at an OA referral centre. The local Ethics Committee approved the study (Hospital Valld’Hebron Ethics Committee approval PR(AG)26/2006). All subjects were contacted specifically to be included in this study and they signed informed consent documents for participation.

### Subjects

All subjects (n = 78) with final diagnosis of OA after a positive specific inhalation challenge (SIC) between January 2008 and December 2013 were included. Medical charts of all subjects were reviewed by the authors. Demographic data such as sex, age, smoking habit, atopy, dermatitis, rhinitis, conjunctivitis, type of employment, agents, exposures, time between start of exposure and start of symptoms, time between start of symptoms and diagnosis, time subjects were away from work until diagnosis, treatment, and severity of asthma according to the Global Initiative for Asthma (GINA) control classification [13] at diagnosis were recorded. Asthma severity was defined in accordance with GINA classification, on the basis of the intensity of treatment required to achieve good control of the condition. Asthma which was well controlled with low intensity treatment such as low-dose inhaled corticosteroids (IC), leukotriene modifiers or chromones was defined as mild. Asthma requiring high intensity treatment to maintain good control, or in which good control was not achieved despite high intensity treatment, was defined as severe. Blood analysis results, including eosinophil count and total IgE, spirometry, methacholine and SIC were assessed.

### Atopy and smoking status

Patients were considered atopic if they had at least one positive prick test to any common environmental allergen. Non-smokers were patients who had never smoked and ex-smokers were those who had not smoked for at least six months. The number of pack-years was calculated.

### Spirometry and methacholine challenge

Spirometry was performed with a Datospir 200 (Sibel, Barcelona) instrument, following the European Respiratory Society (ERS) and American Thoracic Society (ATS) guidelines [14]. The reference values used were those proposed for the Mediterranean population [15]. Bronchial challenge with methacholine was performed in accordance with Spanish guidelines [16]. Briefly, a Mefar MB3 (Mefar, Ele H2O, Medicalli, Brescia, Italy) dosimeter was used, and increasing concentrations of methacholine (from 0.03 mg/ml to 16 mg/ml) were inhaled at three-minute intervals until FEV1 had fallen by 20% compared with its baseline value or until the subject had inhaled the maximum concentration of methacholine. The provocative concentration of methacholine causing a 20% drop in FEV1 was designated as PC20 and expressed in mg/ml. The methacholine challenge was considered negative if the PC20 was higher than 16 mg/ml. In all patients, the methacholine dose-response ratio (DRR) was calculated as the percentage fall in FEV1 at the last concentration, divided by the total concentration administered.

### Specific Inhalation challenge

SIC was carried out according to the guidelines proposed by our group [17]. Briefly, subjects were examined on five consecutive days. Inhaled corticosteroids were withheld 48 hours before SIC. On the first day (control day), full medical and occupational histories were collected, and skin-prick tests with a battery of common allergens, radiography study, pulmonary function testing and methacholine challenge were performed. On day 2, a first placebo inhalation challenge was performed. On days 3 and 4, subjects underwent SIC with the suspected workplace agent. On day 5, pulmonary function test and methacholine challenge were repeated. Changes in lung function were monitored in each patient by measuring FEV1 every 10 minutes during the first hour after exposure and then every hour until 15 hours after inhalation. Response was considered positive when FEV_1_ fell more than 20% compared with the baseline value in the absence of any change to placebo. Asthma response was defined as early when the fall in FEV1 occurred within 1 h of the last inhalation of the sensitizing agent, and as late when the fall in FEV1 was observed between 2–8 h following the challenge. Finally, a combination of an early and late response was defined as a “dual asthma response”.

### Statistical analysis

The characteristics of the subjects are expressed as the median and range unless otherwise stated. A one-sample Kolmogorov-Smirnov test, calculated to assess normality, showed non-normal distribution of the parameters studied. Between-group differences were analysed by the Mann-Whitney test and within-group differences by the Wilcoxon signed rank test. Differences were considered significant at a p value of ≤0.05. Multivariate logistic regression was used to analyse the independent association between asthma severity and the type of agent involved. All variables that were related to the quantities of interest and/or factors previously reported in the literature were considered as potential confounders. Results were reported using odds ratios (OR) and 95% confidence intervals (CI). SPSS release 17.0 for Windows (SPSS; Chicago, IL) and GraphPad InStat4 (GraphPad Software Inc; San Diego, CA) were used for the statistical analyses.

## Results

Of the 78 patients with final diagnosis of OA, 23 responded to HMW agents and 55 to LMW agents. The various sensitizing agents responsible for OA are shown in Table 1. In the group sensitized to HMW agents, flour was the more prevalent (48% of cases). For LMW agents, isocyanates and persulphates were the most prevalent (36% and 24% of cases respectively). Subjects’ demographic data are summarized in Table 2. Although the percentage of patients with atopy was similar in the two groups, patients with OA caused by HMW more frequently presented rhinitis and conjunctivitis than patients with OA caused by LMW.

**Table 1:** Sensitizing agents responsible for OA in the study population.

HMW Agents		LMW Agents	
n = 23		n = 55	
Flour	11	Isocyanates	20
Iroko	2	Persulfate salts	13
Cork dust	2	Welding fumes	3
Ipe	1	Zinc	2
Wood dust mixture	1	Chromium	2
*Plantago ovata*	3	Nickel	1
*Boletus edulis*	1	Colistin	1
Mouse proteins	1	Piperacillin	1
Latex	1	Captopril	1
		Aescina amorfa	1
		Cleaning products	2
		Amines	2
		Cutting fluids	1
		Surfactants	1
		Polyethylene	1
		Epoxyresins	1
		Cyanoacrylate	1
		Rosin	1

Ipe is a hardwood from the Brazilian rainforests

**Table 2:** Demographic characteristics of the study population.

	HMW (23)	LMW (55)	p
**Age, yrs**	40 (23–61)	40 (19–59)	0.86
**Sex, male; n (%)**	17 (74)	28 (51)	0.06
**Smoking habit, n (%)**			0.70
**- Non smoker**	13 (57)	(51)	
**- Smoker**	6 (26)	17 (31)	
**- Ex-smoker**	4 (17)	10 (18)	
**Atopy, n (%)**	9 (39)	17 (31)	0.50
**Dermatitis, n (%)**	7 (34)	12 (22)	0.42
**Rhinitis, n (%)**	20 (87)	33 (60)	**0.02**
**Conjunctivitis, n (%)**	12 (52)	15 (27)	**0.04**
**Total IgE, kU/L**	88 (9–709)	71 (6–797)	0.41
**% blood eosinophils**	4 (1–7)	3 (0–12)	0.61
**FVC, % predicted**	88 (55–118)	88 (71–127)	0.86
**FEV1, % predicted**	90 (53–118)	88 (64–131)	0.82
**FEV1%**	78 (65–93)	78 (58–95)	0.77
**Methacholine test**			0.64
**Positive test, n (%)**	17 (74)	38 (69)	
**PC**_**20**_ **mg/ml**	2.8 (0.05–16)	2.2 (0.25–14.6)	
**Methacholine DRR**	5.5 (0–466.67)	2.87 (0.05–92)	0.29

Data are presented as median (range), unless otherwise stated. FVC: Forced Vital Capacity; FEV_1_: forced expiratory volume in one-second; HMW: high-molecular-weight; LMW: low-molecular-weight; PC_20_: concentration of methacholine inducing a 20% fall in FEV_1_; DRR, dose/response ratio; ≠ Only patients with PC_20_ ≤16 mg/mL

Table 3 presents data on the occupational exposure of patients, severity of asthma and the treatment they were receiving at the time of diagnosis. Patients with OA caused by LMW agents seemed to have more severe asthma than those with OA caused by HMW agents and a greater use of long-acting beta-antagonists (LABA), probably related to the greater severity. No differences were seen between the groups in other variables. A logistic regression analysis confirms that patients with OA caused by LMW agents had a significantly higher risk of asthma severity according to the GINA classification after adjusting for potential confounders (OR = 7.16, 95% CI: 1.13–15,20; p = 0.036) (Table 4).

**Table 3:** Data on occupational exposure, asthma severity and treatment received by patients at the time of diagnosis.

	HMW (23)	LMW (55)	p
**Occupation starting age, yrs**	23 (14–52)	22 (15–21)	0.97
**Duration of exposure, months**	122 (22–528)	115 (5–840)	0.88
**Latency between starting work and symptoms, months**	38 (2–516)	68 (0–468)	0.45
**Latency between symptoms and diagnosis, months**	59 (2–360)	48 (1–504)	0.58
**Latency between last exposure and SIC, months**	0 (0–23)	0 (0–15)	0.56
**Asthma severity, n (%)**			**0.02**
**Intermittent or mild persistent**	17 (74)	25 (46)	
**Moderate or severe persistent**	6 (26)	30 (54)	
IC dose, mcg/day*	200 (0–800)	200 (0–800)	0.35
**n (%)**	14 (61)	32 (58)	0,96
**IC + LABA, n (%)**	6 (26)	30 (54)	**0.02**
**Antileukotrienes, n (%)**	3 (13)	9 (16)	0.71
**Antihistamínes, n (%)**	5 (22)	4 (7)	0.07
Emergency visits**	1 (0–10)	2 (0–15)	0.44
Hospitalizations**	0 (0–2)	0 (0–2)	0.65

Data are presented as median (range), unless otherwise stated. HMW–High molecular weight agents; LMW–Low molecular weight agents; LABA: Long-acting beta-agonists; IC–inhaled corticosteroids; SIC—Specific inhalation challenge.

* Equivalent dose budesonide

** Number of visits per patient in the last 10 years

**Table 4:** Logistic regression analysis with patients exposed to HMW agents as independent variable comparing patients with intermittent or mild asthma and those with moderate or severe asthma.

	OR	95% CI	p
**Agent (LMW)**	7.16	1.13–15.20	**0.036**
**Smoking status**			
**Never**	1		
**Former**	2.70	0.80–9.21	0.11
**Current**	1.48	0.40–5.44	0.52
**Rhinitis**	3.72	0.80–17.40	0.094
**Age of onset of occupation (years)**	1.04	0.98–1.10	0.27
**PC20**	1.02	0.91–1.14	0.70
**Duration of exposure**	0.95	0.87–1.04	0.25
**Latency between starting work and symptoms**	1.01	0.99–1.07	0.75

LMW–Low molecular weight agents; OR–Odds Ratio; CI–Confidence interval.

Finally, Table 5 shows the results in the SIC, suggesting that HMW agents typically induced an early reaction, whereas LMW typically induced a delayed reaction (p = 0.0001). In patients with OA caused by HMW agents SIC was positive with a shorter exposure time (p = 0.025) and these patients required more rescue medication during the SIC (p = 0.003) than those with OA caused by LMW agents. However, patients with OA caused by LMW agents presented a greater degree of bronchial hyperresponsiveness after the SIC, measured as the difference in the values of methacholine DRR (p = 0.024). No differences were found in the fall in FEV1, regardless of the type of response (early, late or dual) or type of agent. Considering the population as a whole, patients requiring treatment with IC alone had a greater decrease in FEV1 after SIC than those taking IC + LABA; median (range): 27.5 (16. 61) and 21 (15–48), respectively, p = 0.005. These differences are not observed when the population is divided into LMW and HMW groups.

**Table 5:** Results of specific inhalation challenge.

	HMW	LMW	p
	n = 23	n = 55	
**Type of reaction, n (%)**			**0.0001**
**Early**	19 (82)	11 (20)	
**Late**	2 (9)	40 (73)	
**Dual**	2 (9)	4 (7)	
**% fall in FEV1**			0.09
**Early**	27 (20–50)	29 (21–47)	
**Late**	19 (15–30)	21.5 (15–50)	
**Dual**	43 (25–61)	28 (25–42)	
**Methacholine test 24h after exposure, n**	14	37	
**Positive test, n (%)**	10 (71)	30 (81)	0.46
**PC**_**20,**_ **mg/mL ≠**	2.5 (0.1–7.3)	2.51 (0.3–16)	0.28
**PC**_**20**_ **decrease ≥2 fold, n (%)≠**	4 (40)	11 (37)	0.69
**DRR methacholine 24 h after exposure, n**	14	37	
**DRR**	3.81 (0–144)	6.15 (0–80)	0.79
**DRR increase ≥2 fold, n (%)**	4 (28)	14 (38)	0.19
**Difference in DRR (pre-post SIC)**	0.87 (0–72)	1.77 (0–16)	**0.02**
**Time of exposure SIC, min**	7 (1–60)	15 (1–120)	**0.02**
**Use of medication during SIC, Yes / No**	6 / 17	2 / 53	**0.003**
**FEV1 decrease during SIC**			
**No treatment**	23 (18–61)	21 (15–42)	0.10
**IC treatment**	28 (22–61)	25.5 (16–50)	0.23
**IC + LABA treatment**	28.5 (25–50)	25 (15–50)	0.21

Data are presented as median (range), unless otherwise stated. HMW–High molecular weight agents; LMW–Low molecular weight agents; DRR–Dose Response ratio; IC–Inhaled corticosteroids; SIC–Specific inhalation challenge; **≠** Only patients with PC_20_ ≤16 mg/mL;. Early asthmatic response: defined when the fall in FEV1 occurred within 1 h of the last inhalation of the sensitizing agent; Late asthmatic response: defined when the fall in FEV1 was observed between 2–8 h following the challenge; Dual asthmatic response: defined as the combination of an early and late asthmatic response.

## Discussion

To our knowledge, this is the first study to show that OA produced by LMW agents may be more severe than that produced by HMW agents. In fact, few studies have assessed the severity of OA at the time of diagnosis, even though it is acknowledged that OA in general may be a particularly severe form of the disease, for three main reasons: 1) the asthma persists in all patients with OA who remain in contact with the causal agent and worsens in 50% in spite of treatment; likewise, it may persist in 50% of patients even though they avoid exposure, and may worsen in 10–40% [18–21]; 2) there have been reports of patients who died after developing acute asthma after occupational exposure to agents to which they were sensitized [22–23]; and 3) patients with OA and WEA consume ten times more medical resources than patients with non-work-related asthma [24]. Other reasons that may explain why OA may be more severe than non-OA are the difficulty of diagnosis, the difficulty of management, generally higher levels of exposure (peaks) in the workplace than in other environments, co-exposures to irritants, and so on.

Interestingly, in the Epidemiological Study on the Genetics and Environment of Asthma (EGEA study) of patients with severe asthma, Le Moual et al [25] found that up to 30% may be exposed to OA-causing agents and that the condition may be more severe in those who are exposed simultaneously to both HMW and LMW agents. The recent observation that persistent occupational exposure to asthmagens (either HMW or LMW) is associated with uncontrolled adult-onset asthma [26] or more severe forms of the disease [25, 27] supports this hypothesis. Furthermore, a longer latency between onset of symptoms and diagnosis of OA carries a worse prognosis for persistence and severity of asthma, whether or not exposure to the causative agent is avoided [28–29]. However, in our study it does not seem that either the total exposure time, or the latency between onset of symptoms and diagnosis were associated with the greater severity recorded in LMW-related asthma. In fact, in agreement with Dufour et al [11], we did not find any differences in the latency period before the onset of symptoms or in the duration of exposure between HMW and LMW agents. In this regard, several authors have pointed out that the length of time necessary for sensitization may depend, among other factors such as genetics or the concentration of inhaled agents [30], upon the nature of each agent rather than on the molecular weight of the agent alone. In this sense, in contrast to the results found in our study, Descatha et al [31], comparing the characteristics of patients with OA to HMW and LMW agents, found that the severity of the disease at the time of diagnosis does not appear to be influenced by the molecular weight of the causal agent.

In the present study, we did not find any association that might explain why OA is more severe when caused by LMW agents. A plausible hypothesis is that agents with different mechanisms of action may trigger different responses in terms of both inflammation and bronchial hyperresponsiveness, thus altering the degree of severity. In line with other authors [10–11, 17] we found that in the context of the challenge test, HMW agents tend to present an early airway response, while in the case of LMW the response is usually late, although early reactions can occur with LMW agents and late reactions with HMW agents. It is generally accepted that HMW agents cause asthma through an IgE-mediated mechanism, that is, via a Th2 response, and generate a clearly eosinophilic airway inflammation; they are also associated with a higher proportion of patients with rhinitis and conjunctivitis [6], as we observed in the present study. In this regard, Malo et al [32] found that the prevalence of symptoms did not differ for HMW and LMW agents, although rhinitis was more intense for HMW than for LMW. Likewise, it is recognized that eosinophilic asthma generally responds well to treatment with IC [13].

The situation is the reverse in the case of patients with OA caused by LMW agents. In fact, the pathogenesis of OA caused by LMW agents remains largely unclear. The data available suggest that the T-cell subsets and cytokine profiles involved in LMW-induced OA may differ from those operating in atopic asthma. Although some of them induce IgE-mediated responses [6], most induce asthma through a non-IgE related mechanism [33] in which non adaptative immune responses might play a role [34]. The possible role of non-immunological mechanisms such as epithelial injury, remodeling of the airway wall, oxidative stress or neurogenic inflammation are under debate [35]. This means that although the inflammation is eosinophilic in some patients, in many others it is neutrophilic or mixed, and in these cases the response to IC treatment is lower [35], these patients may require more treatment and their condition may therefore be classified as more severe [13].

Finally, another interesting result of this study is the observation that individuals with OA caused by LMW agents present greater bronchial hyperresponsiveness 24 hours after the SIC. In this sense, Vandenplas et al [36] demonstrated that SIC to LMW agents is the principal risk factor for the occurrence of asthmatic reactions requiring administration of short-acting beta agonists with or without oral or intravenous corticosteroids. We cannot rule out the possibility that different intrinsic mechanisms may be at work in thepathogenesis of OA caused by HMW or LMW agents. The IgE-mediated response characteristic of HMW agents causes a histamine release which in turn leads to a fall in FEV1 and also, since it is an isolated exposure, a return to baseline levels within a short period of time, which may mean that the degree of bronchial hyperresponsiveness remains unchanged. In OA caused by LMW agents, on the other hand, in addition to possible inflammatory mechanisms, the bronchial hyperresponsiveness may depend on a neuroimmune interaction involving both mast cell activation and the transient receptor potential ankyrin (TRPA)1-dependent stimulation of sensory neurons [37].

Without doubt, the main limitation of this study is its retrospective nature. We do not have objective measures of the degree of asthma control in our patients and so we cannot be sure that the classification of asthma severity at the time of diagnosis was correct. However, the data (including the treatment required by patients) were recorded at the time the SIC was conducted. It is essential that asthma is controlled before performing SIC, because otherwise the results may be misinterpreted and false positives may be obtained [17]. Prior to the SIC, asthma control is usually established by checking that there are no clinical changes or changes in pulmonary function after administration of a placebo [38]. None of our patients presented any such alterations and all underwent the SIC, so it can probably be assumed that their disease was controlled and that the severity was correctly classified. Another limitation is the small number of participants. We can not rule out the possibility that other variables might have reached statistically significant values with a larger number of observations. Finally, some authors have suggested that the outcome of OA varies according to geographical location [29]. This study was conducted in a European country, in which IC are widely used in the treatment of asthma–a practice which may alter the natural history of the disease [39].

In conclusion, this study demonstrates that OA caused by LMW agents may be more severe than that caused by HMW agents. However, the characteristics of the study do not allow us to draw any conclusions about the prognosis of the disease, especially since no differences were found in the baseline lung function, emergency visits or hospitalizations between patients with OA exposed to HMW or LMW agents.The confirmation that most LMW agents induce a delayed response and HMW agents an early response in the SIC, and the differences in the degree of bronchial hyperresponsiveness after the challenge, suggest that the two types of agent have different mechanisms of action. Equally, the absence of any variables associated with the increased severity caused by LMW agents in the present study suggests that these different mechanisms of action are also responsible for the severity of OA. Future studies with larger study populations are necessary to confirm these findings.
